# Prevalence of sleep disturbances and associated factors among Chinese residents: A web-based empirical survey of 2019

**DOI:** 10.7189/jogh.13.04071

**Published:** 2023-08-04

**Authors:** Jing Wang, Jianxiong Wu, Jiaming Liu, Yuan Meng, Jinxi Li, Pengfei Zhou, Minzhi Xu, Qin Yan, Qinnan Li, Xiaoxv Yin, Yanhong Gong

**Affiliations:** Department of Social Medicine and Health Management, School of Public Health, Tongji Medical College, Huazhong University of Science and Technology, Wuhan, Hubei, China

## Abstract

**Background:**

To identify the prevalence of sleep disorders in China through a large sample study. To explore the relevant social determinants affecting residents’ sleep status at both individual and provincial levels based on the theoretical framework of the Dahlgren-Whitehead model.

**Methods:**

A nationwide cross-sectional web-based survey was conducted from January 20 to February 28, 2019 across 31 provinces of China. The Pittsburgh Sleep Quality Index was used to evaluate residents’ sleep quality. Multilevel linear regression analysis was used to analyse the influencing factors of sleep disorder.

**Results:**

A sample of 107 650 residents completed the survey, and 94 454 questionnaires were included in the final analysis. The crude incidence rate and the age-adjusted rate of sleep disorder in Chinese residents were 19.16% and 21.25%, respectively. Those who were older, female, smokers, drinkers, married, divorced, or widowed, retired, more educated (regression coefficient (b) = 0.172, *P* < 0.05), had worse self-perceived economic status, and lived far away from community health services (b = 0.758, *P* < 0.05) were more likely to have sleep problems. Physical exercise, social support (b = -1.705, *P* < 0.05), and greening coverage of residential areas (b = -1.769, *P* < 0.05) were protective factors for residents’ sleep quality.

**Conclusions:**

Sleep disorders are prevalent in the Chinese population, with varying incidence rates across provinces. To improve sleep quality, the Chinese government and health management departments should pay more attention to vulnerable groups and promote healthy lifestyles through education. Additionally, the social network can be utilized to provide social support. Improving the ecological environment and daily living environment is also essential.

With the rapid development of the modern economy and society, the social and natural environment is also undergoing rapid changes, and people’s lifestyles are subsequently transformed. Sleep disturbances have become an important public health problem worldwide affecting people’s physical and mental health. There is established epidemiological evidence that inadequate sleep duration and poor sleep quality are associated with premature death and a variety of adverse health outcomes [1,2]. Other than damage to individual health, poor sleep quality also leads to more medical errors, industrial accidents, and traffic jams, which cause huge socioeconomic losses. It was estimated that the social cost of inadequate sleep in the US in 2020 ranged from about 299 to 433 billion US dollars (US$) [1]. Studies showed that the prevalence of sleep disturbances in the general population ranges from 3.9% to 45.0% worldwide [3,4]. There is a large variation between countries and regions. Therefore, it is necessary to conduct relevant studies in different countries and regions to have a more comprehensive picture of the prevalence of sleep disorders worldwide and analyse their associated factors, to provide empirical data to improve the quality of sleep in the public.

Studies on sleep quality related factors have shown that many individual-level factors such as social demographic characteristics, lifestyle habits, and so on have an impact on the occurrence of sleep disorders [5]. Sleep disorders were more common in women and older adults [3]. Smoking and drinking were risk factors for sleep quality. Social support and social structural factors (such as low education level, poor economic status, etc.) were also closely related to the occurrence of sleep disorders, and the sleep status of the population and its influencing factors differed across cultures [3]. These different levels of the association have been demonstrated in many developed countries, however, evidence of the burden of sleep problems from low-and middle-income countries is still lacking [3].

In the past three decades, China’s economic and social transformation has accelerated. In the context of industrialisation and urbanisation, a series of challenges such as population aging, disease spectrum changes, ecological environment, and lifestyle changes have emerged. Factors threatening the health of Chinese residents have gradually increased. Identifying the adverse and changeable risk factors is a prerequisite to effectively improving the sleep and health status of the Chinese population. Currently, the results of regional studies indicate that the current situation of sleep disorders in China is serious [6-8]. But there is a lack of national sleep quality surveys conducted for Chinese residents. And existing studies on the influencing factors of sleep disturbances in China mostly focus on the individual level, neglecting the exploration of macro-social factors. Further research is needed. Besides, although studies related to the social and environmental determinants of sleep have gradually increased since 2010, there are still few studies that systematically analyse and sort out the multidimensional factors of sleep quality based on scientific theoretical models.

Therefore, based on previous studies, the following research hypotheses were proposed for this study: the sleep quality of the Chinese general population is not optimistic, and it is significantly associated with various factors such as individual characteristics, behavioural lifestyle, social network factors, social structural factors, and macro-social factors and so on.

## METHODS

### Ethics approval

This study was approved by the Medical Ethics Committee of Shihezi University (2018-129-01). All participants were informed of the purpose of this study and participated voluntarily in this survey. This study did not involve any personally identifiable information.

### Research design

From January 20 to February 28, 2019, a nationwide cross-sectional web-based study was conducted across 31 provinces in China. To encourage students to participate in actual public health surveys and pay more attention to health issues, Hainan Medical College organizes an annual community survey for students. Population sleep quality is one of the themes of the 2019 Community Survey. We recruited 1955 undergraduate students as investigators at Hainan Medical College and trained them uniformly to conduct online surveys at the community of their own families. With the coordination of community workers, convenience sampling was used to recruit the participants, students sent the link to the questionnaire to community residents by social network. Target respondents were Chinese residents over 15 years old who could read and write in Chinese and could operate a smartphone. Before formally starting the survey, all participants were required to read an electronic informed consent form, which included organizations, purpose, an anonymity guarantee, and a question: Do you agree to participate in this survey (yes / no)? Only residents who chose “yes” can begin filling out the questionnaire by visiting the link. All questionnaires were completed online and submitted anonymously.

### Sample size calculation

This study used the formula for calculating the sample size of the cross-sectional study: 





where the *n* is the sample size; *P* is the hypothesized sleep disturbances proportion of the respondents; *Z_α_* is the normal deviation, the significance level was set to α = 0.05, the *Z_α/2_* = 1.96; δ is the permissible error, it was set to 0.1 *P*. According to the existing literature, the prevalence of sleep disturbances in the general population ranges from 3.90 to 45.00% [3,4] worldwide, while the prevalence in different regions of China ranges from 8.96 to 49.90% [7,9]. To ensure an adequate sample size, we choose the lower rate for the calculation. We assumed that 8.96% of the residents experienced sleep disturbances. The sample size of this study was initially calculated to be 3904. Additionally, a study on sample size estimation suggested that the study design efficiency value should be considered during the sample size calculation for a sample survey. The efficiency value of the research design is the ratio of the variance of the planned sampling method estimator to the variance of the simple random sampling method estimator when the survey unit is the same for the same target quantity [10]. In this study, we assumed the research design efficiency value of the convenience sampling was 3, the required sample size was 11 712 (3904*3). Considering the possibility of non-response in the actual online survey, we found many excellent web-based survey articles with questionnaire completion rates ranging from 40 to 68% [11-14]. We assumed the response rate of this study was 40%, and the ideal sample size was 29280.

### Measurement indicators

The outcome of interest was sleeping status. The Chinese version of the Pittsburgh Sleep Quality Index (PSQI) was used in the present study to evaluate residents’ sleep quality. The scale was translated and validated by Liu et al. in 1996 and was widely used in China [15]. This scale consists of seven dimensions and 18 items. It evaluates multiple dimensions of sleep over one month. These seven dimensions include subjective sleep quality, sleep latency, sleep duration, habitual sleep efficiency, sleep disturbances, use of sleeping medications, and daytime dysfunction. Each component is scored according to the levels 0-3. The accumulative score of each component is the total score of the PSQI. Total scores thus ranged from 0 to 21, where the higher the score, the worse the sleep quality. This study set 7 points as the critical score to calculate the incidence of sleep disturbances [15]. The PSQI has a good reliability coefficient (Cronbach’s alpha) of 0.70 for its seven components.

This study was guided by the theoretical framework of the Dahlgren-Whitehead model [16] to explore the social determinants of sleep disturbances in mainland Chinese residents. The following covariates were collected using single questionnaire entries or data searching: Individual-level variables included age (continuous variable), gender (male or female), smoking behavior (smokers or non-smokers), drinking behavior (drinkers or non-drinkers), exercise (1-2, 3-5 or 6-7 times / week), marital status (unmarried, married, divorced or widowed), social support (low, medium or high), work (no job, employed or get retired), education (primary school or below, high school or below, associate degree or above), self-perceived economic status (good, fair or bad), length of time to walk from home to the nearest community health service (within 15-minute, 15-30-minute or over 30-minute).

In addition, we collected macro-level variables by digging into open data like the China Statistical Yearbook 2019, the Main Data of the Seventh National Population Census, and China Ecological and Environmental Bulletin 2018. Macro-social variables such as greening coverage of built-up areas, total dependency ratio, hospital beds (per 1000 population), the percentage of days with good air quality, per capita GDP by province, and the urban registered unemployment rate were included in our study.

### Statistical analysis

Statistical analysis was performed using SAS version 9.2. We calculated the age-standardized prevalence of sleep disturbances in each province to eliminate as much as possible the comparison error caused by differences in age structure. The direct method was used to calculate the age-standardized rate. The standard population was derived from the Main Data of the Seventh National Population Census of China [17]. The data divided the Chinese population into three groups: 0-14 years old, 15-64 years old, and 65 years old and above, accounting for 17.95%, 68.55%, and 13.50% respectively. Since our target population was Chinese residents aged 15 and above, we divided the population into two groups according to the age of census data: 15-64 years old, and 65 years old and above. We then calculated the age-standardized rate of sleep disturbances in each province separately according to the formula: Age-standardized rate of sleep disturbances = ∑ (rate of sleep disturbances in each age group *age composition of the standard population). The descriptive statistical analyses were conducted on the PSQI scores of the respondents with different demographic characteristics in this study, and the results are presented as medians and interquartile ranges (IQRs). Wilcoxon Mann-Whitney tests were conducted to compare the differences in PSQI scores among respondents with different demographic characteristics. Multicollinearity was not founded since the variance inflation factor of these variables was well below the traditional cut-off value of 10. Multilevel linear regression analysis was used to analyze the influencing factors of sleep disorder. For all comparisons, differences were tested using two-tailed tests, and *P* values less than 0.05 were considered statistically significant.

## RESULTS

A total of 107 650 online questionnaires were collected from 31 provinces in China. After removing 13 196 questionnaires with missing key variables, irregular filling, and obvious logic errors, finally, 94 454 questionnaires completed by the respondents were included in the analysis, with an effective rate of 87.74%.

### Prevalence of sleep disorders in China

**Figure 1:**
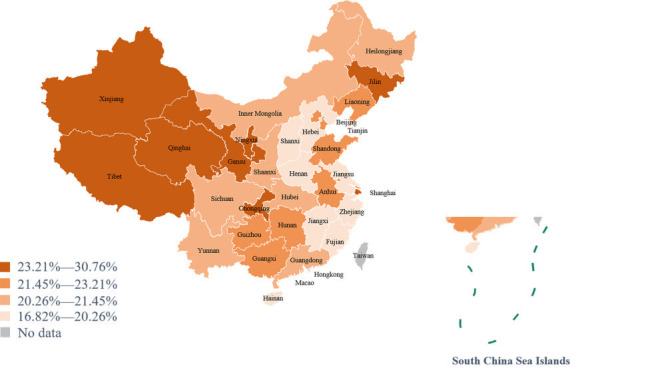
The prevalence of sleep disorders by province in China. Sleep quality data were obtained from all provinces in the mainland of China except Hong Kong, Macau and Taiwan. The darker the color, the higher the prevalence of sleep disturbances.

Of the 94 454 completed questionnaires, the average PSQI score was 4.96 ± 3.14. 18 101 participants had sleep disorders. The crude incidence rate and the age-adjusted rate of sleep disorder were 19.16% and 21.25%, respectively. The prevalence of sleep disorders differs in different provinces. The three provinces with the highest age-adjusted rate were Qinghai, Xinjiang, and Shanghai (30.75%, 28.21%, and 26.32%, respectively). The three provinces with the lowest age-adjusted rate were Zhejiang, Jiangsu, and Jiangxi (18.42%, 17.00%, and 16.83%, respectively). The specific prevalence of sleep disorders was shown in **Figure 1**.

### Individual-level comparison of sleep quality among subjects with different characteristics

**Table 1** presents the sleep quality scores of the population with different demographic characteristics. Participants’ sleep quality was significantly different by all individual-level factors at *P* < 0.05. In this study, residents who were female, smokers, alcohol drinkers, who lacked physical exercises, who were divorced or widowed, who lacked social support, who were retired, who had lower education levels, who reported worse economic status, and who had poor access to health services had higher PSQI scores. The main sleep symptoms of the 18 101 residents with sleep disorders were having difficulty falling asleep (16 242, 90.27%) and easily awakening during the night or in the early morning (13 620, 75.70%). The details are shown in **Table 2**.

**Table 1:** Sleep quality scores of the participants with different demographic characteristics

	Score of PSQI			
	**Medians**	**P25**	**P75**	P*
Gender				<0.05
*Male*	4.00	3.00	7.00	
*Female*	5.00	3.00	7.00	
Smoking behaviour				<0.05
*Non-smokers*	4.00	3.00	6.00	
*Smokers*	5.00	3.00	8.00	
Drinking behaviour				<0.05
*Non-drinkers*	4.00	2.00	6.00	
*Drinkers*	5.00	3.00	7.00	
Frequency of physical exercise				<0.05
*No*	5.00	3.00	7.00	
*1-2 times / week*	4.00	3.00	6.00	
*3-5 times / week*	4.00	2.00	6.00	
*6-7 times / week*	4.00	2.00	7.00	
Marital status				<0.05
*Unmarried*	4.00	3.00	7.00	
*Married*	4.00	3.00	7.00	
*Divorced*	6.00	3.00	9.00	
*Widowed*	6.00	4.00	9.00	
Social support				<0.05
*Low*	6.00	3.00	9.00	
*Medium*	5.00	3.00	7.00	
*High*	4.00	2.00	6.00	
Work type				<0.05
*No job*	4.00	3.00	7.00	
*Employed*	4.00	3.00	7.00	
*Get retired*	5.00	3.00	8.00	
Education level				<0.05
*Primary school or below*	5.00	3.00	8.00	
*High school or below*	5.00	3.00	7.00	
*Associate degree or above*	4.00	3.00	7.00	
Self-perceived economic status				<0.05
*Good*	4.00	2.00	6.00	
*Fair*	4.00	3.00	6.00	
*Bad*	5.00	3.00	7.00	
Length of time to walk from home to the nearest community health service				<0.05
*Within 15 min*	4.00	2.00	6.00	
*15-30 min*	5.00	3.00	7.00	
*Over 30 min*	5.00	3.00	8.00	

PSQI – the Pittsburgh Sleep Quality Index, P25 – lower quartile, P75 – upper quartile

*Kolmogorov-Smirnov test was used to examine the normality of the distribution of continuous variables. The *P* value is associated with Wilcoxon Mann-Whitney test.

**Table 2:** Main symptoms of the residents with sleep disturbances (n = 17 992)

Symptoms*	n	%
Cannot get to sleep within 30 min	16 341	90.28
Wake up in the middle of the night or early morning	13 708	75.73
Have to get up to use the bathroom	11 713	64.71
Cannot breathe comfortably	7332	40.51
Cough or snore loudly	9126	50.42
Feel too cold	11 401	62.99
Feel too hot	9266	51.19
Had bad dreams	11 212	61.94
Have pain	8904	49.19
Other reasons	12 122	66.97

*This table presents 10 specific manifestations of sleep disturbances.

### Multilevel linear regression analysis: The association of PSQI score with social determinants of health at individual and provincial levels

**Table 3** shows the results of a two-level multilevel linear regression analysis. Model 1 included variables for individual factors (age and gender). Based on model 1, health behaviors were added to model 2, including smoking, drinking, and physical exercise. Compared with model 2, social network factors were added to model 3, including marriage, social support, and job type. Compared with model 3, social structural factors were added to model 4, including education level, self-perceived economic status, and health service accessibility. Model 5 included all variables. In addition to the individual level variables, macro-social variables at the provincial level were also added to Model 5, including greening coverage of built-up areas, total dependency ratio, hospital beds (per 1000 population), the percentage of days with good air quality, per capita GDP by province, and urban registered unemployment rate. Any reduction in the AIC was an improvement in the fit of the model. It showed that model 5 had the best fit, so we chose model 5 as the final model.

**Table 3:** Estimates from two-level linear regression models predicting PSQI

Variables	Model 1	Model 2	Model 3	Model 4	Model 5
Intercept	4.322*	3.960*	5.236*	4.152*	5.106*
Age	0.017*	0.016*	0.012*	0.013*	0.013*
Gender (Ref = male)					
*Female*	0.191*	0.624*	0.639*	0.643*	0.642*
Smoking behavior (Ref = non-smokers)					
*Smokers*		0.900*	0.807*	0.790*	0.789*
Drinking behavior (Ref = non-drinkers)					
*Drinkers*		0.660*	0.651*	0.629*	0.628*
Frequency of physical exercise (Ref = no)					
*1-2 times / week*		-0.512*	-0.447*	-0.360*	-0.361*
*3-5 times / week*		-0.617*	-0.521*	-0.403*	-0.403*
*6-7 times / week*		-0.690*	-0.634*	-0.529*	-0.529*
Marital status (Ref = unmarried)					
*Married*			0.033	0.162*	0.162*
*Divorced*			0.844*	0.914*	0.914*
*Widowed*			0.992*	1.011*	1.011*
Social support (Ref = low)					
*Medium*			-0.840*	-0.854*	-0.854*
*High*			-1.771*	-1.706*	-1.706*
Work type (Ref = no job)					
*Employed*			0.018	0.133*	0.133*
*Retired*			0.375*	0.480*	0.480*
Education level (Ref = primary school or below)					
*High school or below*				0.084	0.085
*Associate degree or above*				0.177†	0.177†
Self-perceived economic status (Ref = good)					
*Fair*				0.380*	0.380*
*Bad*				1.016*	1.015*
Length of time to walk from home to the nearest community health service (Ref = within 15 min)					
*15-30 minutes*				0.483*	0.482*
*Over 30 minutes*				0.756*	0.755*
Greening of built-up areas					-2.868‡
Total dependency ratio					0.565
Hospital beds (per 1000 population)					0.002
Percentage of days with good air quality					0.085
Per capita GDP by province					0.019
Urban registered unemployment rate					-8.517
Model fit					
*AIC*	483305.8	480166.9	477417.3	475347.9	475343.0

PSQI – the Pittsburgh Sleep Quality Index, Ref – reference, GDP – gross domestic product, AIC – Akaike information criterion

*P < 0.001.

†*P* < 0.01.

‡*P* < 0.05.

Model 5 showed that for individual variables, people with older age and women had higher PSQI scores. As for health behaviours, smoking (regression coefficient (b) = 0.789, *P* < 0.05) and drinking (b = 0.630, *P* < 0.05) were the risk factors for sleep quality, while physical exercise (b = -0.527, *P* < 0.05) was a protective factor for residents’ sleep status. Residents’ PSQI scores decreased as the number of exercises per week increased. As for social and community networks factors, compared with unmarried residents, married, divorced, and widowed individuals all had the risk of increased PSQI scores. Social support was a protective factor for sleep quality. The higher the level of social support, the lower the PSQI score of residents. Those who were employed had higher PSQI scores compared to those who had no jobs. As for social structural factors, residents who had associate degrees or higher, who reported bad economic status, and who lived far away from community health service institution had higher PSQI scores. As for macro-social variables, high greening coverage was a protective factor for sleep quality.

## DISCUSSION

In this cross-sectional survey of 94 182 China general residents, we found that sleep disturbances were common among Chinese residents. Multiple social determinants at five levels, including individual characteristics, behavioural lifestyle, community network factors, social structural factors, and macro-social factors, were significantly associated with sleep quality, which was consistent with our previous research hypothesis.

This study showed that nearly one-fifth (19.16%) of the participants had poor sleep quality. Compared with domestic studies that also use the PSQI scale with a cut-off value of 7, the results of this study are quite different from those of studies conducted in other regions of China. Surveys conducted in Xuzhou City [7] and Liaoning Province [6] in 2013 showed that the incidence of sleep disorders among residents over 18 years old was 8.96% and 11.59%, respectively. Data from Ya’an, Sichuan Province in 2021 showed that the incidence of sleep disorders among urban residents was 31.60% [18]. This may be related to the differences in survey population, time, and regions. A national community survey conducted in Japan showed that 32.70% of residents had sleep disorders [19]. The incidence of this health problem in Spain is 38.2% [20]. The differences in the results of these studies may be related to the different cut-off values for the psychometric properties of the scale appropriate for different populations[21]. This limited the comparability of the results of this study with other studies to some extent. In general, sleep disorders were common in the Chinese population. More attention should be paid by Chinese health administration and academia.

Based on the theoretical framework of Dahlgren-Whitehead model, this study systematically reviewed the social determinants of sleep disorders among Chinese residents. The innermost layer of the model is the individual characteristic factor, which is the inherent individual feature determined by biological genetic factors that cannot be changed. In the present study, women and older residents had a higher risk of sleep disturbances, and these findings have been well-validated in previous studies [19,22-25]. The second layer is modifiable lifestyle behaviour. Many health-related behaviors directly affect the population’s health status [26]. This study confirmed that behaviors such as smoking and drinking were risk factors for sleep disorders, while regular exercise could help improve sleep quality. This may be because nicotine, as a harmful stimulant, affects the activity of the nervous system and causes the brain to be in a constant state of arousal, which makes it difficult to fall asleep or reduces the quality of sleep [24,27]. Studies have shown that long-term smoking can lead to inflammation and narrowing of the respiratory system, which can lead to sleep apnoea and have a negative impact on sleep [27]. As for alcohol, it shortens the duration of deep sleep. Alcohol and its metabolites can cause neurological damage, which can affect the quality of sleep [28]. Regular exercise can stimulate the body to produce melatonin, which is conducive to the regulation of the body’s circadian rhythm. And exercise can consume body energy, so that the muscles are in a relaxed state, thus helping to improve the quality of sleep [29]. The third layer is social network factors. Community resources and social relations possessed by individuals can provide social support for individuals, which will have an impact on their own health. This study found that higher levels of social support were associated with better sleep quality [30]. Divorced and widowed residents had poorer sleep quality than unmarried residents, which is consistent with the findings of a multinational study that included data from 16 countries [22]. The possible explanation is that the breakdown or absence of intimacy may increase the risk of sleep problems by negatively affecting mood and leading to endocrine and autonomic dysregulation [31]. The fourth layer is social structural factors, including socioeconomic status, work environment, health care services, etc. Previous studies found that socioeconomic status was associated with sleep quality and individual health [8,32,33], possibly because it affected the quality of life and sleep status of the residents by influencing individual material conditions [5]. However, in this study, having higher education (associate degree or above) was significantly associated with an increased risk of sleep disorders. We speculated that this might be because highly educated residents were engaged in social positions that required more mental work. Although the health information and skills possessed by residents with a high intellectual level of knowledge can prevent health problems by influencing their lifestyle behaviors. With the improvement of the general knowledge level of residents and the rapid development of modern office equipment, the demands on the knowledge and technical level of the mental workforce are also increasing in all jobs, leading to occupational tension. This continuous mental pressure will interfere with sleep quality [34,35]. Besides, the results of this study suggested that residents with high accessibility to health services had relatively better sleep quality. It might be because these residents were closer to the community health service center. They could receive more timely treatment when health problems arose, thus having relatively higher-level health and promoting their sleep quality. The outermost layer is macro-social factors, including the social and natural environment. This study found that greening coverage of residential areas was a protective factor for residents’ sleep quality. It was consistent with a national survey in the Netherlands [36]. Green space could significantly alleviate the perceived life stress among respondents. Different departments, such as urban planning and health management, should value this positive impact. Strengthen cross-sectoral cooperation to promote urban greening and improve the overall quality of life of residents.

This study is the largest survey on the sleep quality of residents in China, covering 30 provinces in mainland China, which has good representation.

This study is one of the few empirical studies to apply the theory of social determinants of health to the field of sleep disorders. The theoretical framework of the Dahlgren-Whitehead model has the following two advantages: First, it provides a relatively comprehensive theoretical model. It is helpful for us to systematically tease out the relevant social determinants affecting the sleep status of Chinese residents at both individual and provincial levels. The results of this study identify potentially effective health policy intervention points for improving the sleep quality of Chinese residents. The exploration of influencing factors at different levels will help define opportunities for cross-sectoral cooperation. Second, this study demonstrates the feasibility of the theoretical framework of the Dahlgren-Whitehead model in a general Chinese population. Social determinants of health may have different effects on different populations due to the different social cultures and internal structures of health systems in different regions of different countries. Therefore, there is currently little empirical evidence on the social determinants of sleep disorders in the general population of developing countries [37]. This empirical study provides a guideline for future research on sleep health. The theoretical framework of the Dahlgren-Whitehead model can help to identify the relevant social determinants of health more comprehensively.

There are several limitations. First, this study was conducted through a web-based questionnaire, leading to a large number of young people who are better at using mobile phones, which may underestimate the incidence of sleep disorders. The present study was able to compensate for this limitation to some extent by standardizing the age. Second, this study was based on an online questionnaire survey to assess Chinese residents’ sleep problems. This method could not necessarily reflect the actual sleep pattern problems directly. The actual sleep pattern problems need to rely on further clinical observation and diagnosis. Further studies need to consider combining clinical diagnosis to better assess sleep pattern problems. Moreover, except for the factors investigated in this study, there are other related factors that could affect sleep. Considering the loss of residents’ patience in filling out the questionnaire and the richness of the questionnaire content, we did not investigate too many influencing factors to ensure the questionnaire filling quality as high was as possible. The relevant factors that were not identified in this study needed to be further explored in subsequent studies

## CONCLUSIONS

This study surveyed the sleep disturbances of the general population in China through a web-based questionnaire and explored the association between social determinants of health and sleep disturbances. This study found that the prevalence of sleep disorders among Chinese residents was at a high level. And the detection rate of sleep disorders in different provinces was significantly different. Attention should be given to women, the elderly and low-income groups, etc. Publicity and education related to a healthy lifestyle should be expanded to guide residents to form a healthy lifestyle in line with their characteristics. In addition, the relevant departments should strengthen public health services and urban greening to cover the whole population, improve the capacity of health services, and modify the ecological environment and economic and social development patterns. So as to relieve the psychological pressure on residents, improve sleep quality and achieve a sound and coordinated development of health, economy, and society.
